# Electromagnetic fields exposure on fetal and childhood abnormalities: Systematic review and meta-analysis

**DOI:** 10.1515/med-2023-0697

**Published:** 2023-05-12

**Authors:** Zahra Atarodi Kashani, Reza Pakzad, Farzaneh Rashidi Fakari, Mohammad Sadegh Haghparast, Fatemeh Abdi, Zohreh Kiani, Afsaneh Talebi, Somaieh Moradi Haghgoo

**Affiliations:** Department of Nursing and Midwifery, Iranshahr University of Medical Sciences, Iranshahr, Iran; Students Research Committee, Ilam University of Medical Sciences, Ilam, Iran; Department of Midwifery, School of Medicine, North Khorasan University of Medical Sciences, Bojnurd, Iran; School of Medicine, Shahid Beheshti University of Medical Sciences, Tehran, Iran; Non-communicable Diseases Research Center, Alborz University of Medical Sciences, Karaj, Iran; Faculty of Nursing and Midwifery, Ahvaz Jundishapur University of Medical Sciences and Health Services, Ahvas, Iran; Department of Midwifery, School of Nursing and Midwifery, Ahvaz Jundishapur University of Medical Sciences, Ahvaz, Iran; Students Research Center, Hamadan University of Medical Sciences, Hamadan, Iran

**Keywords:** childhood abnormalities, electromagnetic fields, fetal diseases

## Abstract

Today, in the modern world, people are often exposed to electromagnetic waves, which can have undesirable effects on cell components that lead to differentiation and abnormalities in cell proliferation, deoxyribonucleic acid (DNA) damage, chromosomal abnormalities, cancers, and birth defects. This study aimed to investigate the effect of electromagnetic waves on fetal and childhood abnormalities. PubMed, Scopus, Web of Science, ProQuest, Cochrane Library, and Google Scholar were searched on 1 January 2023. The Cochran’s *Q*-test and *I* ^2^ statistics were applied to assess heterogeneity, a random-effects model was used to estimate the pooled odds ratio (OR), standardized mean difference (SMD), and mean difference for different outcomes, and a meta-regression method was utilized to investigate the factors affecting heterogeneity between studies. A total of 14 studies were included in the analysis, and the outcomes investigated were: change in gene expression, oxidant parameters, antioxidant parameters, and DNA damage parameters in the umbilical cord blood of the fetus and fetal developmental disorders, cancers, and childhood development disorders. Totally, the events of fetal and childhood abnormalities were more common in parents who have been exposed to EMFs compared to those who have not (SMD and 95% confidence interval [CI], 0.25 [0.15–0.35]; *I* ^2^, 91%). Moreover, fetal developmental disorders (OR, 1.34; CI, 1.17–1.52; *I* ^2^, 0%); cancer (OR, 1.14; CI, 1.05–1.23; *I* ^2^, 60.1%); childhood development disorders (OR, 2.10; CI, 1.00–3.21; *I* ^2^, 0%); changes in gene expression (mean difference [MD], 1.02; CI, 0.67–1.37; *I* ^2^, 93%); oxidant parameters (MD, 0.94; CI, 0.70–1.18; *I* ^2^, 61.3%); and DNA damage parameters (MD, 1.01; CI, 0.17–1.86; *I* ^2^, 91.6%) in parents who have been exposed to EMFs were more than those in parents who have not. According to meta-regression, publication year has a significant effect on heterogeneity (coefficient: 0.033; 0.009–0.057). Maternal exposure to electromagnetic fields, especially in the first trimester of pregnancy, due to the high level of stem cells and their high sensitivity to this radiation, the biochemical parameters of the umbilical cord blood examined was shown increased oxidative stress reactions, changes in protein gene expression, DNA damage, and increased embryonic abnormalities. In addition, parental exposure to ionizing and non-ionizing radiation can lead to the enhancement of different cell-based cancers and developmental disorders such as speech problems in childhood.

## 1 Introduction

Congenital malformations are known as deformations and chromosomal abnormalities. Congenital anomalies include behavioral, structural, metabolic, and functional disorders in infants [1]. These disorders can be diagnosed before or after birth. Congenital anomalies are one of the global health problems. Each year, 8 million babies (6% of all births worldwide) are born with a severe birth defect. At least 3.3 million children between the ages of 0–5 years die from severe birth defects, and each year approximately 300,000 infants die of congenital defects within the first 28 days of life [2,3].

In developed countries, approximately 30% of deaths in children (less than 5 years old) are due to congenital malformations [4]. The results of a study in England and Wales showed that out of 628,171 gross births (stillbirths and live births), a total of 13,400 children were born with one or more congenital anomalies. That is, out of every 47 births (stillbirths and live births), there is one case of congenital abnormalities. The results of the same ecological study in the UK showed that the prevalence of hospital admission due to congenital anomalies has increased significantly from 1999 to 2019 (19.6%) [1]. In addition, the results of another Australian study from 2005 to 2015 reported an increasing trend in admissions due to congenital anomalies [5]. One of the most important risk factors for congenital abnormalities is the age of the mother, which increases the risk of chromosomal abnormalities such as Down syndrome [1].

According to the National Congenital Anomaly and Rare Disease Registration Service report, the rate of congenital anomalies in the 35–39 year age group (229.9 per thousand live births) is significantly higher than in the 30–34 and 25–29 year age groups (187.1 and 192.2 per thousand live births, respectively) [6]. In addition to maternal age, low income may be one of the indirect causes of congenital anomalies. About 94% of severe congenital anomalies occur in low- and middle-income countries. Related to this issue, in low- and middle-income countries, pregnant women are more prone to malnutrition, reduced access to screening and health care, and environmental pollutants [7]. Congenital malformations may be the result of one or a combination of socioeconomic factors (low income), demographics, genetics (gene mutations), maternal infections (such as syphilis and rubella), maternal nutritional status (such as folate deficiency), or environmental teratogens. The reasons for congenital abnormalities are complex and multifactorial, but in most cases, their etiology is unknown. Most congenital abnormalities are caused by complex interactions between genes and the environment that are largely unknown [8,9]. In the modern world, most populations are exposed to EMF radiation [10], and public concern about the potential health hazards of extremely low-frequency-electromagnetic fields (ELF-EMFs) powers and radio frequency (RF)/microwave radiation emissions has increased [11].

EMFs are non-ionic radiations that cannot release electrons. In fact, energy is in the form of electric oscillations and magnetic fields that are transferred from one point to another [12]. According to frequency, EMF can be classified into four different types. The first type refers to extremely low frequency, which is below 300 Hz and is generated by military equipment, railways, and high-voltage power lines. The second type is known as intermediate frequency EMFs, which are in the range of 300 Hz to 10 MHz and are produced by industrial cables and electrical equipment in homes, such as televisions and computers. The third type of EMFs is High Frequency, with frequencies in the range of 10 MHz to 3 GHz, which are produced by mobile phones and radios. Radio frequencies, which have a maximum frequency of 100 MHz, are also included in this category. Moreover, static EMFs are produced by magnetic resonance imaging (MRI) and geomagnetism and are determined by zero frequency [13]. Another type of classification based on wavelength and resonance is classified into categories, such as (radio and TV, microwaves, infrared, visible light, ultraviolet, X-rays, and gamma rays). Radio waves contain any electromagnetic wave produced by currents in wires and circuits. RF is divided into subcategories, such as microwaves and electromagnetic waves used for amplitude modulation (AM) and frequency modulation radio, cellular telephones, and TV. The lowest radio frequencies are produced by high-voltage AC power transmission lines at frequencies of 50 or 60 Hz and extremely long wavelength electromagnetic waves (about 6,000 km! approximately) [14,15]. All people are exposed to these two types of EMFs: (a) EMFs from electrical and electronic devices and power lines and (b) RF radiation from wireless devices such as cordless phones, cell towers, antennas, and transmission towers that broadcast [11].

EMFs have a high penetration power and the ability to move charged particles such as electrons and large macromolecule ions and polymers [16]. Therefore, with high concentrations of electrons and ions, they can have destructive effects on tissue [17]. Magnetic fields can have several different effects, such as differentiation and abnormalities in cell proliferation, damaged deoxyribonucleic acid (DNA) and chromosome abnormalities, blood disorders, and congenital defects in cell components [18,19,20,21]. Through exposure to magnetic fields that produce currents and electric fields, these waves can disorder the body’s physiological balance, increase the lifespan of free radicals, and lead to DNA damage in individuals [22]. In addition, according to gender, density of body tissue, life cycle, and exposure, the effect of environmental pollution varies. Many sources can cause humans to be exposed to magnetic fields. Power supplies, computers, televisions, radios, and telephones are some of these resources. Recently, it has been shown that electromagnetic waves emitted from phones lead to oxidative stress in human semen. Keeping the phone in a pocket of trousers while talking may negatively affect sperm and impair male fertility [23]. Cell phones emit a type of radiofrequency radiation called radio waves [24]. Daily exposure to radio waves has increased concerns about infertility, stillbirth, congenital anomalies, and abortions [25,26]. Although some researchers have reported findings on undesirable fertility outcomes, no specific abnormalities or other undesirable outcomes have been consistently reported. Of course, most studies have limited statistical power [27].

Some findings reveal that human exposure to radiofrequency (RF) waves or living near high-voltage power lines can cause cognitive and behavioral disorders, reduced learning and memory power, and poor neurobehavioral function [28]. Significant heat effects from the waves are associated with adverse health outcomes such as sleep problems, hearing problems, reproduction problems, nervous system disorders, and increased cancer [29]. In this regard, children and adults who live near high-voltage towers or lines are more prone to develop acute lymphocytic leukemia (ALL) and neurodegenerative disorders such as Alzheimer’s and Parkinson’s, respectively [30,31]. Exposure to RF-EMF during pregnancy can also affect fetal growth and the duration of the pregnancy. This effect can occur as a result of changes in maternal physiology either directly or indirectly through radiation to the fetus [32]. However, there is no definitive proof that these radio frequency radiation (RFR) systems (Wi-Fi and mobile phones) are harmful or not to humans [33]. MRI is applied as an essential tool in investigating various diseases, and its use has increased during pregnancy. There is not enough information about the consequences of exposure to magnetic fields on the fetus [32].

The main purpose of this study was to answer the question of whether maternal exposure to non-ionizing radiation (such as electromagnetic fields [EMFs] or RFR), as well as maternal exposure to ionizing radiation (X-ray examinations) during pregnancy, is related to fetal and childhood developmental disorders and the risk of cancer in childhood. In general, the results of studies in this field are contradictory. The results of some studies on maternal exposure during pregnancy to ELF-EMFs (more than 50 Hz or 0.82 mG) or RF-EMFs (place of residence at a distance of less than 2 km) showed that the risk of congenital malformations or fetal [34,35,36,37], childhood developmental disorders [38], and the possibility of cancer increased [39]. On the contrary, the results of some studies did not show a significant increase in terms of the risk of fetal developmental disorders and the possibility of childhood cancer [40]. The results of some studies on the exposure of mothers to ionizing radiation during pregnancy, especially in the first trimester, showed a significant increase in the risk of rhabdomyosarcoma [41] and brain tumors, such as primitive neuroectodermal tumor during childhood offspring’s [42].

Sometimes physicians are asked to determine whether exposure to radiation before birth can pose a risk to the development of the fetus or not [43] and whether lack of awareness of the consequences of exposure to radiation can cause unreasonable concern [44,45]. Physicians who care for pregnant mothers often overestimate the teratogenic danger associated with diagnostic imaging [46]. Therefore, awareness of their potential effects leads to appropriate advice and desirable decision-making. According to contradictory data, no systematic study has been done to investigate the effect of electromagnetic waves on fetal and child abnormalities.

## 2 Methods

The guidelines of Preferred Reporting Items for Systematic Review and Meta-Analysis (PRISMA) were followed while reporting the study protocol [47,48]. In addition, in accordance with the PRISMA guidelines, the following steps were taken: a systematic literature search, organization of documents for the review, abstracting and quality assessment of each empirical study, synthesizing data, and writing the report. The protocol of the study was registered in the International Prospective Register of Systematic Reviews (PROSPERO) at the National Institute for Health Research. Registration Number in PROSPERO is CRD42021235681.

### 2.1 Search strategy

The search strategy described below is applied based on Population, Exposure, Comparison and Outcome (PECO) for MEDLINE and then used in other databases. PubMed, Scopus, Web of Science, ProQuest, Cochrane systematic review were systematically searched up to 1 January 2023. The text words and Medical Subject Headings (MeSH) terms Electromagnetic Fields Exposure and Childhood Abnormalities were used to search. The PECO in our study was as follows:

Population: Childhood or Fetal

Exposure: Exposure to Electromagnetic Fields

Comparison: Non-exposure to Electromagnetic Fields

Outcome: Fetal and Childhood Abnormalities

The search strategy is described below, is applied based on PECO for MEDLINE (MeSH), and then used in other databases:

1. Childhood (title/abstract) OR Childhood (Mesh term)
2. Fetal (title/abstract) OR Fetal (Mesh term)
3. Fetus (title/abstract) OR Fetus (Mesh term)
4. Embryonic (text word) OR Embryonic (Mesh term)
5. 1 OR 2 OR 3 OR 4
6. Electromagnetic fields (title/abstract) OR electromagnetic fields (Mesh term)
7. Electromagnetic radiation (title/abstract) OR electromagnetic radiation (Mesh term)
8. Electromagnetic wave (title/abstract) OR electromagnetic wave (Mesh term)
9. Electromagnetic energy (title/abstract) OR electromagnetic energy (Mesh term)
10. Cell phone (title/abstract) OR Cell phone (Mesh term)
11. 6 OR 7 OR 8 OR 9 OR 10
12. Fetal disorder (title/abstract) OR fetal disorder (Mesh term)
13. Embryonic disorder (title/abstract) OR embryonic disorder (Mesh term)
14. Childhood abnormality (title/abstract) OR childhood abnormality (Mesh term)
15. Childhood disorder (title/abstract) OR childhood disorder (Mesh term)
16. 12 OR 13 OR 14 OR 15
17. 5 AND 11 AND 16

More detail of search strategy was provided in Box A1.

### 2.2 Inclusion and exclusion criteria

Observational studies conducted up to 1 January 2023 were included in this review. Letters, comments, and case reports were excluded. There are no language restrictions on using and entering articles in this study. If the language used in an article is other than Persian or English, we asked a translator to translate the article.

The criteria for admission include women for whom intrauterine pregnancy, single pregnancy, and spontaneous pregnancy without the use of assisted technologies have been confirmed. The criteria for exclusion include studies in which pregnant women have a history of chronic diseases such as diabetes, hypertension, cardiovascular disorders, genetic disorders, birth defects in previous pregnancies, and smoking.

### 2.3 Study selection

**Figure 1:**
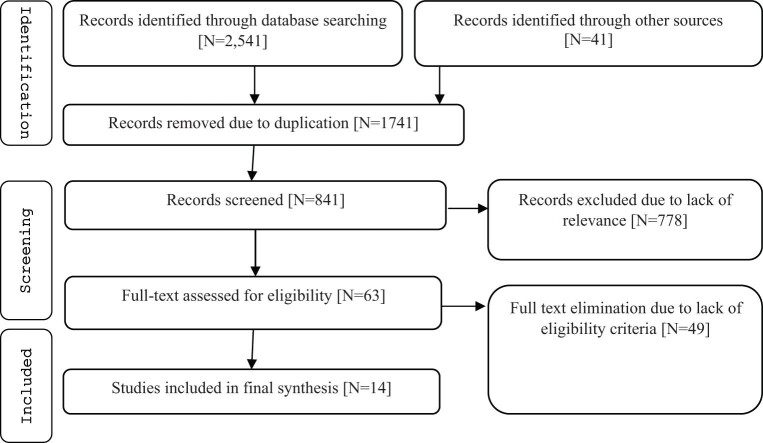
PRISMA flowchart of selected studies.

The initial search in five databases resulted in 2,541 studies. Also, 41 studies were found from other sources like expert opinion and gray literature. After removing duplicated papers, further 841 papers were screened based on title and abstract. Finally, 63 papers appeared to be potentially eligible, and their full-texts were reviewed. In this process, 14 studies met the eligibility criteria and were included in the meta-analysis. Figure 1 shows the search process based on the PRISMA flowchart.

### 2.4 Quality assessment

The quality of each study was assessed according to the Newcastle–Ottawa scale (NOS) [49]. A maximum of ten stars can be given to each study based on the NOS. NOS scoring for cross-sectional study included: very good: 8–10 stars, good: 6–7 stars, satisfactory: 4–5 stars and unsatisfactory: 3–0 stars. NOS scoring for cohort and case-control studies included: very good: 7–9 stars, good: 5–6 stars, satisfactory: 4 stars and unsatisfactory: 3–0 stars [50,51].

### 2.5 Data extraction

Two of the authors independently selected the studies and validity assessment by using a researcher-made form which included: the first author’s name, publication year, study design, sample size, exposed and unexposed group-based type device producer electromagnetic field, outcome was examined and quality score resolved any disagreements by consulting a third researcher. In any disagreements between the two authors, a third researcher resolved the discrepancy by consulting (Table 1).

**Table 1:** Overview of all studies included in the systematic review and meta-analysis

Author [ref]	Year	Region	Study design	Sample size	Participants	Type of EMF signal	Exposure	Outcome		Effect size	QAS
Su et al. [35]	2014	China	Cross-sectional	149	Pregnant women in first trimester who were seeking induced abortion	Power-line MFs	Maternal exposure to magnetic fields ≥0.82 mG and <0.82 mG	Embryonic sac length ≤25th percentile (20 mm) (*N* = 36); >25th percentile (*N* = 94)		OR: 1.56 (0.7–3.48)	5
								Embryonic bud length ≤25th percentile (7 mm) (*N* = 19); >25th percentile (*N* = 46)		OR: 3.95 (1.10–14.20)	
Mahram and Ghazavi [11]	2013	Iran	Cohort	380	Pregnant women and their newborns	High-voltage power lines	Maternal exposure during pregnancy to high-voltage power lines or living at a distance of 25 m from cables or high-voltage towers (mean MF: 3.0104 ± 1,081 mG) *N* = 222 vs unexposed group (mean MF: 0.419 ± 0.04 mG) *N* = 158	Congenital malformation		1.43 (0.35 to 5.83)	6
Sadeghi et al. [37]	2017	Iran	Nested case–control	304	Case: the mothers with spontaneous preterm childbirth (*N* = 152); control: the mothers with tern childbirth	High-voltage power lines or statin	Maternal exposure during pregnancy to high-voltage power lines or living at a distance of 600 m from cables or high voltage towers *N* = 28 (9.8%) vs unexposed group (≥600 m) *N* = 257 (90.2%)	Congenital malformation		5.05 (1.52–16.78)	
Blaasaas et al. [34]	2002	Norway	Population-based study	836,475	Records of the pregnant women that registered in Medical Birth of Norway	Occupational exposure to 50 Hz EMF (melting industry, welders, machinists, pilots, some occupations in textile industries, woodworking factories, working with electricity, glass, and ceramics)	Occupational exposure maternity to magnetic fields above 0.1 μT ≥ 24 h/week vs < 4 h/week) (0.1 µT = 1 mG)	Case: all CNS defect (*N* = 1,171); control group (*N* = 777,039)		OR: 1.41 (0.81–2.44)	6
								Case: spina bifida (*N* = 409): control group (*N* = 777,801)		OR: 2.33 (1.10–4.94)	
Tabrizi and Bidgoli [30]	2015	Iran	Case–control	122	Children diagnose with ALL and normal children <12 years	Residence near high-voltage power lines	Neonatal and childhood exposure to high voltage power lines (>4 yeras) by questionnaire	Case group: childhood ALL (*N* = 22); control group: healthy children (*N* = 100)		3.65 (1.69–7.87)	4
Zarei et al. [38]	2019	Iran	Case–control	185	Mothers of children aged 3–7 year diagnosed with speech problems and mothers of healthy children	Residence near high-voltage power lines	Maternal exposure to high-tension power line before pregnancy by questionnaire	Case group: children with speech problems (*N* = 110); control group: healthy children (*N* = 75)		18.96 (1.11–325.49)	5
							Maternal exposure to high-tension power lines during pregnancy by questionnaire			4.34 (1.22–15.38)	
Lerman et al. [36]	2001	Israel	Case–control	434 Women with 933 pregnancies	Female physiotherapists who had ever been pregnant	Occupational exposure to 27.12 MHz EMF (shortwaves and ultrasonic device)	Occupational exposure of physiotherapist mothers to shortwaves device vs unexposed group	Case: congenital malformation (*N* = 41); control group (*N* = 512)		2.24 (1.28–4.83)	5
							Occupational exposure of physiotherapist mothers to ultrasonic device vs unexposed group	Case: congenital malformation (*N* = 40); control group (*N* = 633)		3.23 (1.5–8.68)	
Hug et al. [40]	2010	German	Case–control	4,431	Children 0–14 years old with and without childhood cancer	Occupational exposures: metal workers, electric welders, locomotive engineers, and power plant operators	Fathers’ pre-conception exposure to EMFs ≤0.2 μT and >0.2 μT (0.2 µT = 2 mG)	Case: children with cancer (*N* = 2,049); control group: healthy children (*N* = 2,382)		1.03 (0.83–1.18)	6
Ha et al. [39]	2007	South Korean	Case–control	1,928	Children under age 15 years with leukemia	Radio transmitter (with a power of 20 kW or more)	Residence address <2 km from the nearest radio transmitter vs who was living >20 km from it	Case: children with leukemia (*N* = 808); control group (*N* = 676)		2.5 (1–4.67)	5
							Q1: <518.41 mV/m	Lymphocytic leukemia (case: 514 vs control: 513)		1	
							Q2: 518.41–<624.35 mV/m			1.39 (1.04–1.86)	
							Q3: 624.35–<916.96 mV/m			1.59 (1.19–2.11)	
							Q4: _916.96 mV/m			1.08 (0.8–1.45)	
Bektas et al. [54]	2020	Turkey	Cohort	149	Pregnant women after birth, placenta and cord blood samples were collected	Use of mobile phone and Wi-Fi (2.4 GHz)	Maternal exposure to mobile phone: Group 1: user mobile phone (*N* = 48), Group 2: Wi-Fi exposed (*N* = 17), Group 3: mobile phone plus Wi-Fi (*N* = 64) vs unexposed group (control) (*N* = 20)	DNA damage was detected in lymphocytes of the samples of cord blood with Comet test	Tail intensity	Control group: 24.72 ± 2.79	5
										Group 1: 46.13 ± 11.50	
										Group 2: 23.76 ± 4.45	
										Group 3: 46.86 ± 11.47 (*p* < 0.001)	
									Tail movement	Control group: 41.32 ± 10.09	
										Group 1: 106.95 ± 71.78	
										Group 2: 38.13 ± 7.87	
										Group 3: 127.82 ± 81.01 (*p* < 0.001)	
								Oxidant factors in cord blood	Protein carbonyl	Control group: 62.54 ± 16.28	
										Group 1: 82.92 ± 17.01	
										Group 2: 68.46 ± 11.93	
										Group 3: 86.68 ± 13.22 (*p* < 0.001)	
									8-Hydroxy-20-deoxyguanosine	Control group: 81.63 ± 9.87	
										Group 1: 96.11 ± 13.84	
										Group 2: 80.43 ± 11.44	
										Group 3: 99.77 ± 15.12 (*p* < 0.001)	
									Malondialdehyde [53]	Control group: 10.35 ± 7.86	
										Group 1: 25.45 ± 15.47	
										Group 2: 11.87 ± 7.55	
										Group 3: 27.48 ± 12.03 (*p* < 0.001)	
									Total oxidant status (TOS)	Control group: 9.08 ± 1.94	
										Group 1: 11.30 ± 2.11	
										Group 2: 10.18 ± 1.86	
										Group 3: 10.96 ± 2.15 (*p* < 0.001)	
								Antioxidant factors in cord blood	TOS	Control group: 1.28 ± 0.48	
										Group 1: 0.89 ± 0.377	
										Group 2: 1.09 ± 0.43	
										Group 3: 0.91 ± 0.41 (*p* < 0.001)	
								Ratio of total oxidant to total antioxidant	Oxidative stress index (OSI)	Control group: 8.29 ± 3.95	
										Group 1: 14.65 ± 5.61	
										Group 2: 10.45 ± 3.67	
										Group 3: 14.62 ± 7.44 (*p* < 0.001)	
Bektas et al. [33]	2018	Turkey	Cohort	149	Pregnant women were divided into three groups based on the duration of mobile phone use per day	Radiofrequencies (RFs) emitted from mobile phones	Mother who user mobile phone: Group 1 (2–15 min/day), *N* = 39, Group 2: (15–60 min/day), *N* = 37, Group 3: more than 60 min/day, *N* = 36; control group: nonusers of mobile phone *N* = 37	Biochemical parameters of cord blood	Aspartate aminotransferase	Control group: 44.54 ± 19.94	4
										Group 1: 46.61 ± 17.17	
										Group 2: 64.83 ± 18.75	
										Group 3: 75.05 ± 23.39 (*p* < 0.001	
									Alanine aminotransferase	Control group: 49.29 ± 28.27	
										Group 1: 42.51 ± 22.28	
										Group 2: 47.81 ± 14.94	
										Group 3: 61.38 ± 31.25, (*p* < 0.001)	
									Lactate dehydrogenase	Control group: 386.75 ± 147.98	
										Group 1: 765.97 ± 483.77	
										Group 2: 807.10 ± 262.77	
										Group 3: 866.91 ± 279.63, (*p* < 0.001	
									Creatine kinase	Control group: 84.18 ± 35.72	
										Group 1: 110.28 ± 95.21	
										Group 2: 172.35 ± 118.73	
										Group 3: 497.88 ± 156.09 (*p* < 0.001)	
									Creatine kinase–myocardial band	Control group: 67.18 ± 40.91	
										Group 1: 81.69 ± 23.85	
										Group 2: 95.00 ± 29.14	
										Group 3: 127.66 ± 49.53 (*p* < 0.001)	
									C-reactive protein	Control group: 0.71 ± 0.46	
										Group 1: 0.80 ± 0.38	
										Group 2: 1.01 ± 0.58	
										Group 3: 3.77 ± 2.03 (*p* < 0.001)	
									Troponin T	Control group: 18.57 ± 4.81	
										Group 1: 18.32 ± 3.54	
										Group 2: 26.16 ± 7.16	
										Group 3: 70.03 ± 15.05 (*p* < 0.001)	
									Uric acid	Control group: 3.04 ± 0.64	
										Group 1: 3.31 ± 0.68	
										Group 2: 3.53 ± 0.80	
										Group 3: 4.72 ± 1.01 (*p* < 0.001)	
									Platelet	Control group: 271955.56 ± 70,826	
										Group 1: 283953.97 ± 82,324	
										Group 2: 278397 ± 64,684	
										Group 3: 241356.77 ± 62,525 (*p* < 0.029)	
Luo et al. [55]	2013	China	Cohort	40	Pregnant women in gestational age: 50–60 day after 20 min exposure to RF-EMF, were undergoing the induced abortion	Electro-magnetic wave emitted from cellular telephone (Global System for Mobile communications network; 900 MHz frequency; maximum power < 1 W; specific absorption rate 1.46 W/kg)	Maternal exposure to EMFs from cell phone (*N* = 20) vs control group: mother not exposure (*N* = 20)	Deferentially expressed proteins profile in chorionic tissues: COMT, Capzb, and antioxidant factor: TXNL-2	Capzb	Control group: 0.67 ± 0.2	6
										Group 1: 1.8 ± 0.23 (*p* < 0.05)	
									COMT	Control group: 0.3 ± 0.25	
										Group 1: 0.7 ± 0.3 (*p* < 0.05)	
									TXNL2	Control group: 1.3 ± 0.35	
										Group 1: 0.9 ± 0.4 (*p* < 0.05)	
Grufferman et al. [41]	2009	USA	Case–control	618 (Case: 311 and control: 309)	Children under the age of 20 years with and without rhabdomyosarcoma and their mothers	X-rays examinations	X-ray examinations in mothers throughout pregnancy vs not examination	Case rhabdomyosarcoma in children (*N* = 311); control group (*N* = 309)		1.9 (1.1–3.4)	6
							X-ray examinations in mothers during first trimester vs not examination	Case: rhabdomyosarcoma in children (*N* = 311); control group (*N* = 309)		5.7 (1.2–27.8)	
Shu et al. [42]	2002	USA	Case–control	3,828 (Case: 1,842 and control: 1,986)	Children under the age of 15 years with and without ALL and their mothers	X-rays examinations	X-ray examinations in mothers throughout pregnancy vs not examination	Case: childhood ALL (*N* = 1,809); control group (*N* = 1,950)		1 (0.8–1.3)	5
							X-ray pelvimetry examinations in mothers throughout pregnancy vs not examination	Case: childhood ALL (*N* = 1,804); control group (*N* = 1,942)		1.2 (0.8–1.7)	
							X-ray examinations of lower abdominal in mothers during preconception vs not examination	Case: childhood T cell ALL (*N* = 181); control group (*N* = 198)		1.2 (0.7–2.1)	
Stålberg et al. [56]	2007	Sweden	Case –control	1,036 (Case: 512 and control: 524)	Children under the age of 15 years with and without Children Brain tumors and subtypes of brain tumor	X-rays examinations	Prenatal abdominal X-ray examination in mothers vs not examination	Case: childhood all brain tumor (*N* = 503); control group (*N* = 512)		1.02 (0.64–1.62)	5
							Prenatal abdominal X-ray examination in mothers vs not examination	Case: primitive neuro ectodermal tumors (*N* = 105); control group (*N* = 512)		1.88 (0.92–3.83)	

### 2.6 Statistical analysis

All analyses were conducted with Stata software version 14.0 (College Station, Texas). For each study, the odds ratio (OR) with 95% confidence interval (CI) or mean and standard deviation of different outcomes were extracted. Since there were two indexes for different outcomes (OR and mean), we defined the “fetal and childhood abnormalities” variable and then converted all indexes to the standardized mean difference (SMD) with 95% CI. For studies that reported OR, it was changed to SMD based on the following formulas:

d={\rm{\log }}({\rm{OR}})\times \frac{\sqrt{3}}{\pi };\hspace{ 1em}{{V}}_{{\rm{d}}}={V}_{{\rm{\log }}({\rm{OR}})}\times \frac{3}{{\pi }^{2}},]

where *d* is SMD, *π* is the 3.14, *V* is variance. A positive SMD means more events of fetal and childhood abnormalities in parents exposed to EMFs, and conversely, a negative SMD means fewer events in exposed parents. Then, for specific outcomes, we calculated pooled OR and pooled mean difference by the “Metan” command. For specific outcomes, OR > 1 and a positive mean difference means more events in exposed parents.

As in previous studies [52,53], heterogeneity was determined using Cochran’s *Q* test of heterogeneity, and the *I* ^2^ index was used to quantify heterogeneity. In accordance with Higgins classification approach, *I* ^2^ values above 0.7 were considered high heterogeneity. To calculate pooled estimation, the fixed-effect model was used, and when the heterogeneity was greater than 0.7, the random effects model was used. The meta-regression analysis was used to examine the effect of sample size, device, and publication date as factors affecting heterogeneity among studies. The “Metabias” command was used to check for publication bias, and if there was any publication bias, the pooled estimation was adjusted with the “Metatrim” command using the trim-and-fill method. In all analyses, a significance level of 0.05 was considered.

## 3 Results

Finally, 14 articles with 854,154 sample sizes were analyzed. The flow chart of the study is shown in Figure 1, and details are shown in Table 1. Studies were published during 2001–2019, and Iran with four studies was a frequent country.

**Figure 2:**
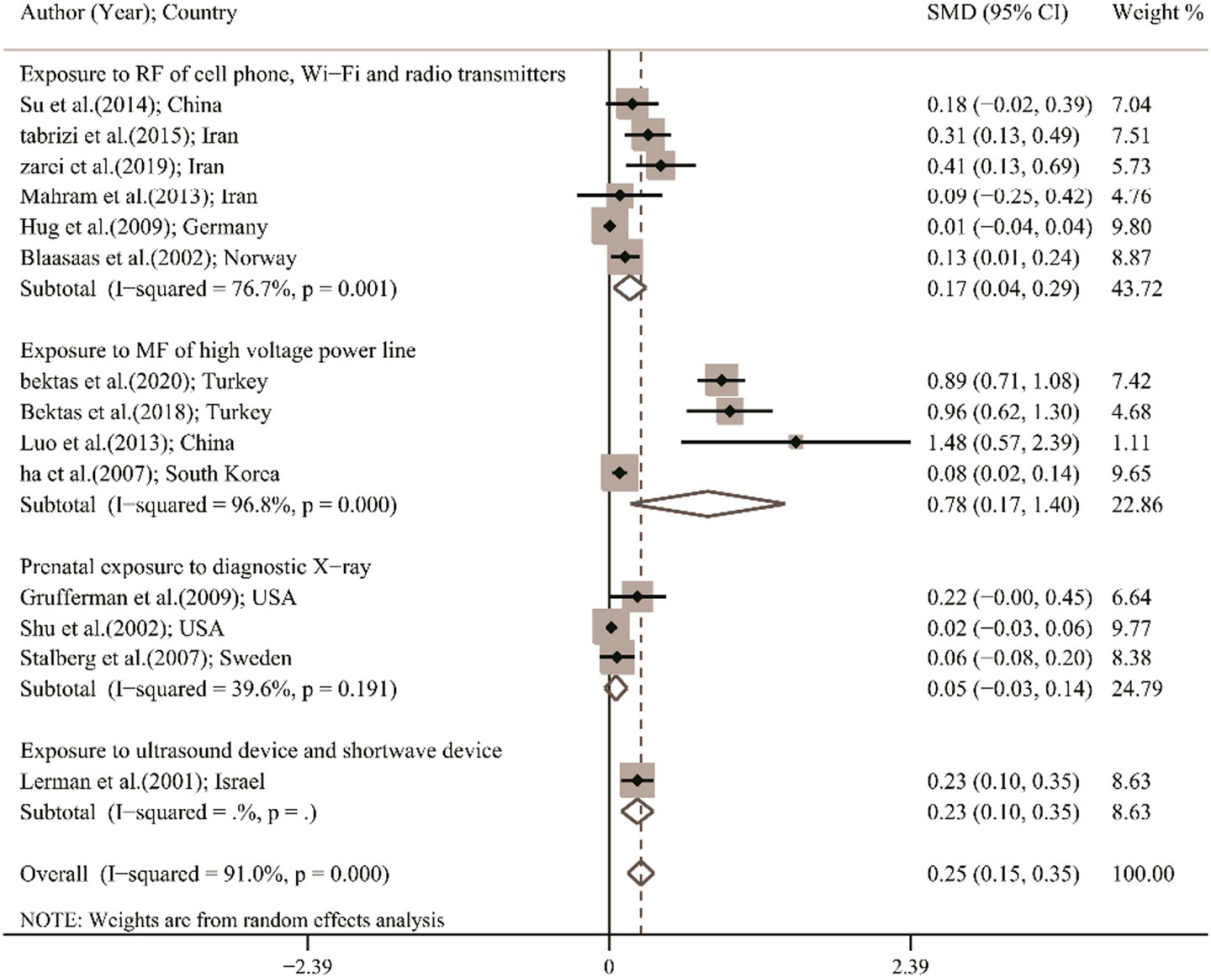
Forest plot of SMD in total and based on different subgroups using a random effects model; the midpoint of each line segment shows estimating the SMD, the length of line segment indicates 95% CI in each study, and diamond mark illustrates the pooled estimate in total and different subgroups.

Figure 2 shows the pooled SMD of fetal and childhood abnormalities. Lowest and highest SMD were observed in the study by Hug et al. in Germany (SMD, 0.01; 95% CI, −0.04 to 0.04) and in the study by Luo et al. in China (SMD, 1.48; 95% CI, 0.57–2.39), respectively. Using a random effect model, the pooled SMD of fetal and childhood abnormalities was 0.25 (95% CI, 0.15–0.35; *I* ^2^, 91%). It means that in fetus and children whose parents have been exposed to EMFs, the event of fetal and childhood abnormalities was higher than in fetus and children whose parents have not been exposed to EMFs.

**Figure 3:**
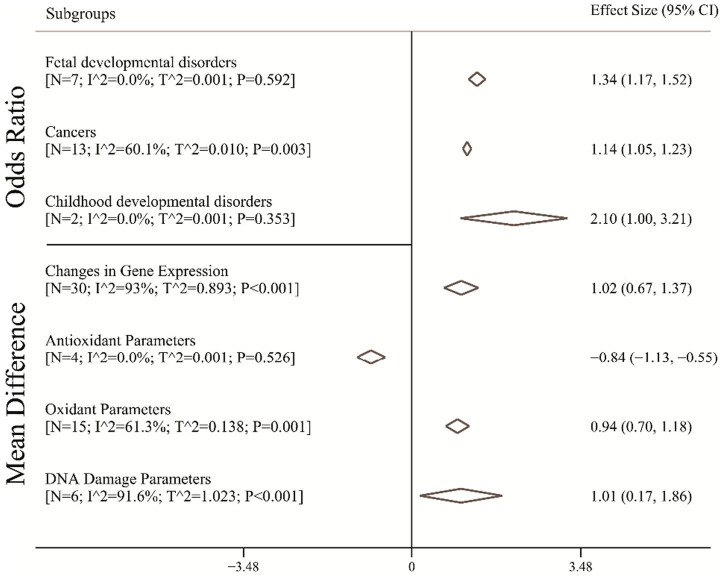
Pooled OR and mean difference with 95% CI of fetal and childhood abnormalities based on the random effects model. The diamond mark illustrates the pooled estimate.

Figure 3 shows the pooled OR and mean difference for different outcomes. The pooled OR for fetal developmental disorders was 1.34 (95% CI, 1.17–1.52; *I* ^2^, 0%). It means that in mothers who have more exposure to EMFs, the odds of fetal developmental disorders in fetus were 1.34 times as many as in mothers who have no exposure to EMFs. Moreover, in parents that have more exposure to EMFs, the odds of cancer in children were 1.14 (95% CI, 1.05–1.23; *I* ^2^, 60.1%) times as many as in parents that have no exposure to EMFs. The pooled OR for development disorders was 2.10 (95% CI, 1.00–3.21; *I* ^2^, 0%). Figure 3 also shows the pooled mean difference for different outcomes. The pooled mean difference of the change in gene expression was 1.02 (95% CI, 0.67–1.37; *I* ^2^, 93%). It means that in mothers who have more exposure to EMFs, the mean change in gene expression in fetus was 1.02 units as many as in mothers who have no exposure to EMFs. In addition, in mothers that have more exposure to EMFs, the mean of antioxidant parameters in fetus was 0.84 (95% CI, −1.13 to −0.55; *I* ^2^, 93%) units lower than in mothers that have no exposure to EMFs. Pooled to EMFs, mean difference of oxidant parameters in fetus cord blood in mothers exposed to EMFs was 0.94 (95% CI, 0.70–1.18; *I* ^2^, 93%) units more than that in fetus in mothers who have no exposure. Finally, the mean of DNA damage parameters in fetus whose mothers were exposed to EMFs was 1.01 (95% CI, 0.17–1.86; *I* ^2^, 91.6%) units more than that fetus whose mothers were not exposed to EMFs.

### 3.1 Heterogeneity, subgroup, and meta-regression analysis

**Figure 4:**
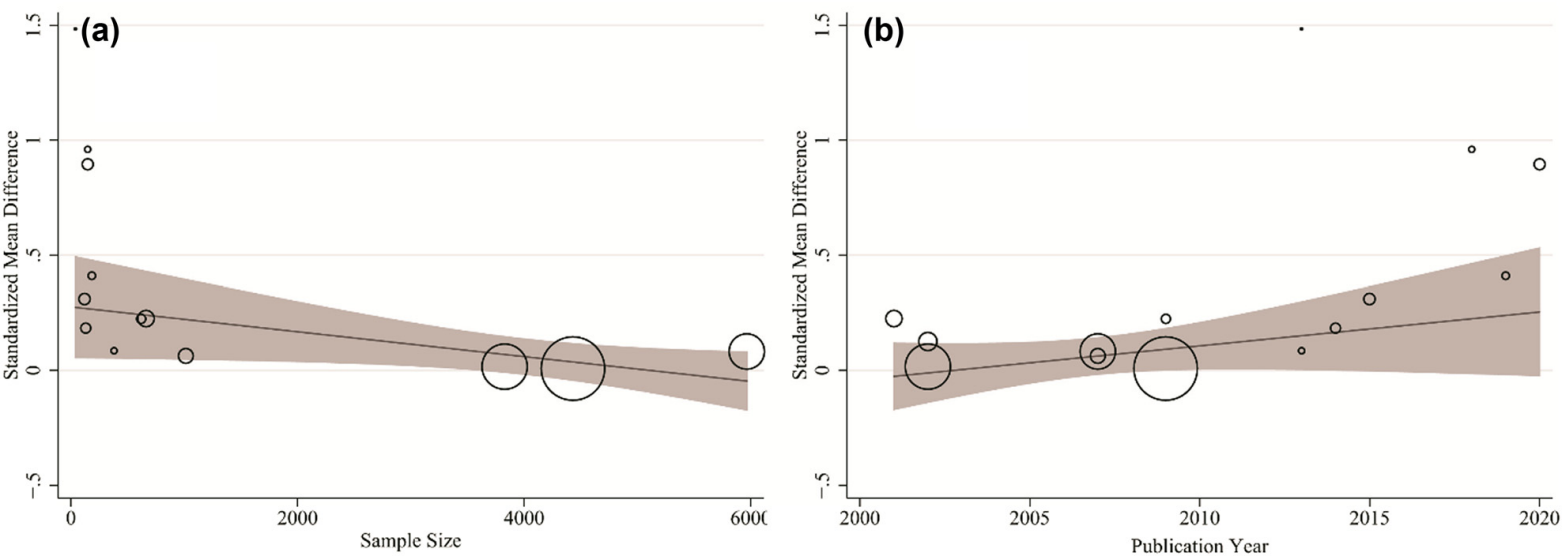
Association between sample size (a) and publication year (b) with SMD of fetal and childhood abnormalities. There was a significant association between publication years and SMD.

As shown in Figure 2, heterogeneity was significant among studies (*I* ^2^, 91%; *p*-value of Cochran’s *Q*-test < 0.001). According to the meta-regression results shown in Table 2, publication year has a significant effect on heterogeneity, so that for 1 year increase in publication year, SMD increased 0.033 (95% CI, 0.009–0.057). This finding is shown in Figure 4b. Sample size (coefficient with 95% CI, −0.001 [−0.002 to 0.001]; Figure 4a) and device (coefficient with 95% CI, −0.001 [−0.023 to 0.021]) have no effects on heterogeneity.

**Table 2:** Results of the univariate meta-regression analysis on the heterogeneity of the determinants

Variables	Sample size	
	Coefficient (95% CI)	*p* value
Publication year	0.033 (0.009 to 0.057)	0.011*
Sample size	−0.001 (−0.002 to 0.001)	0.077
Device	−0.001 (−0.023 to 0.021)	0.940

*: Significance.

Code for device: 1 – exposure to magnetic field of high-voltage power line; 2 – exposure to RF of cell phone, Wi-Fi, and radio transmitters; 3 – prenatal exposure to diagnostic X-ray; and 4 – exposure to ultrasound and shortwave devices.

### 3.2 Publication bias

A significant publication bias was observed for all symptoms as determined by Egger’s test (*Z* score, 2.46; *p* 0.014). Therefore, the fill- and trim-adjusted SMD (0.249; 95% CI, 0.151–0.347) was generated, which was not significantly different from the original SMD (0.25; 95% CI, 0.15–0.35). It means that the result of the meta-analysis was robust.

## 4 Discussion

This study aimed to investigate the effect of EMFs on fetal and childhood disorders if their parents were exposed to them. With the increase in the modernity process and the increase in exposure of mothers to environmental pollutants, the hospital admission rate due to congenital malformations has increased by almost 19% from 1999 to 2019 [1]. The age of the mother above 35 years increases the risk of congenital anomalies by 3.93 times and the risk of spontaneous abortion by 12.82 times [57]. Since that maternal age is a crucial factor in fetal malformations, this risk factor is one of the important risk factors investigated in the screening of aneuploidy tests in the first trimester [58]. The risk of neurodevelopmental disorders in children from assisted reproductive technology is not significantly different from that in children from natural conceptions, except for cerebral palsy, which may be the result of multiple pregnancies or preterm delivery [59]. Even the weight of the fetus for gestational age improves in the frozen embryo transfer method compared to the fresh embryo transfer, which may be due to the suitable environment of the uterus in the frozen embryo transfer method compared to the fresh embryo, but in terms of fetal malformations, there is no significant difference [60]. This shows the importance of the internal environment of the uterus in the process of growth and development of the fetus. According to the developmental origins of health and disease theory, environmental factors affecting health during the fetal period can also affect the health of childhood and adulthood [61]. This relevance has an epigenetic mechanism. Epigenetic mechanism is molecular mechanism that alters gene expression by chemically modifying DNA without affecting the genomic sequence. The embryo and even the egg or sperm cells may be affected by external environments (drugs and environmental pollutants) [62]. Other risk factors for chromosomal disorders and fetal malformations, except maternal age (age over 35 years), include maternal diseases, maternal nutritional deficiency, family or countries with limited resources, and low and middle income countries. This can be due to the increased exposure of the mother to environmental risk factors such as infection, alcohol, limited access to health care, marginalization, exposure to radiation, and certain pollutants. Due to the complex interaction of environment and genetics, most congenital malformations have unknown causes [63].

Through the advancement of technology, humans are exposed to electromagnetic waves generated by various devices. Since exposure to RF and ELF-EMFs overlaps in everyday life, their effects cannot be specifically distinguished; however, this classification is based on the frequency of waves according to the physical definitions, their biological effects are not significantly different, and both of them are non-ionizing rays [64]. If the current density transcends a certain limit, it causes membrane depolarization, unusual changes in calcium ion diffusion, and nerve and muscle stimulation [65]. In addition, radiofrequency (RF) fields generate torques (oscillations) on biomolecules [33]. Since the fetus and infant have large numbers of stem cells that lack adequate immune-mediated sources, environmental pollutions such as the RFR of phones or microwave radiation affect gene expression in stem cells and increase the production of heat shock proteins like HSP70 (these proteins are produced under stress conditions such as oxidative stress) [66].

In this study, the results of the meta-analysis revealed that fetuses and children whose parents were exposed to EMFs had lower mean antioxidant parameters in umbilical cord blood and higher mean umbilical cord oxidant parameters. The results of studies also revealed that RFR 900–1,800 MHz was associated with oxidative stress, DNA damage in the brain and liver, pathological changes in liver tissue, and oxidative damage to the kidney in animal samples [67,68,69,70,71]. In animal cases, exposure to RFR not only increases protein oxidation and DNA damage but also reduces the activity of some antioxidant enzymes [72]. The results of another study revealed that RFR at 834 MHz did not change oxidative stress parameters in the blood and liver tissues of rats [73]. Another study showed that human placental villi, exposed to RF-EMF in the early stages of pregnancy, had lower concentrations of the antioxidant enzyme TXNL-2. A probably decreased amount of placental antioxidants is a key defense mechanism against the biological effects of RF-EMFs in pregnancy [55]. The amount of RFR frequency and the features of the biological substance play an important role in the outcome, so that sensitivity to RFR and ELF-EMFs increases in pregnancy due to dehydration and the presence of a large number of stem cells [74]. Another study in human umbilical cord blood cases revealed that fetuses whose mothers were exposed to RFR emitted from a mobile phone compared to mothers who were only exposed to RFR emitted from Wi-Fi revealed signs of DNA damage, and a significant OSI in these people have increased. It should be noted that the RF waves of a mobile phone are 900, 1,800, or 2,100 MHz, implying that people are closer to the source of the RFR, whereas Wi-Fi devices emit 2.45 GHz RF waves, implying that people are farther away from it. On the other hand, it is revealed that Wi-Fi has had the potential to contribute to the destructive effects of mobile phones [54]. In addition to the importance of maternal distance from the source of RFR production, the duration of exposure or conversation of a person with a cell phone for more than 1 hour during the day is associated with increased biochemical parameters and decreased platelet volume [33].

Exposure of most animal cases and fewer human cases to ELF-EMFs due to fractures and DNA damage is known as a genotoxic agent. For example, it has been revealed that exposure to ELF and RF produces oxidative stress proteins in cells, universal symptoms of distress in plant, animal, and human cells, and DNA damage and neurological effects even at low levels of exposure under current safety standards [15]. The results of the present meta-analysis revealed that fetuses and children whose parents were exposed to EMFs have more gene expression changes and DNA damage. The results of some studies revealed that RFR with oxidative stress leads to the decomposition of structures of biological molecules, such as proteins, lipids, and DNA [67,71] Decreased antioxidant factors and increased oxidative stress parameters in fibrous tissues exposed to RF are associated with damage to proteins and nucleic acids in the placenta that may severely impair normal trophoblastic functions and may even lead to cell death [55].

Exposure to a magnetic field before birth may have an undesirable effect on fetal development. The results of the present meta-analysis study showed that odds increased the chances of developing fetal and childhood developmental disorders in mothers exposed to EMFs more than 1.34 times during pregnancy. Among the studies examined, six studies related the residence near high-voltage power lines with EMFs of more than 1 mG and congenital anomalies, fetal and childhood developmental disorders. The results of the reviewed studies showed that maternal exposure to EMFs significantly increased the OR of developmental disorders in their fetus, such as embryonic bud length less than the 25th percentile (less than 7 mm), 3.95 times and a significant increase in placental apoptosis [35], congenital malformations increased 1.43-fold [11], 5.05-fold [37] and central nervous system (CNS) defects and spina bifida increased 1.43-fold and 2.33-fold, respectively also a significant increase in Clubfoot in the fetus [34]. Animal studies have also shown that pulsed magnetic fields at certain intensities and frequencies may inhibit fetal growth *in vivo* and alter normal cell function [75]. One of the strengths of these studies is the more accurate measurement of mothers’ exposure to EMFs through wearing an EMDEX Lite magnetic field meter for a 24-h period [35] and the accurate determination of the distance between the place of residence and high-voltage power lines using ArcGIS software or determining the duration of occupational exposure to magnetic fields greater than 1 mG [34,37].

Occupational exposure of physiotherapist mothers to a short-wave diathermy and ultrasound device with a frequency 27.12 MHz increases the chances of congenital anomalies by 2,044 times and low birth weight by 2.99 times, and its effect is dose-dependent. Of course, after controlling for confounding factors, there was no significant increase in the rate of congenital anomalies, but the low birth weight was significantly higher, despite the fact that preterm delivery was not significantly different between the two groups [36]. However, the results of another study showed that pregnant women living near high-voltage towers and cables (exposure to ELF-EMFs) did not have a significant effect on gestational age, weight, head circumference, and congenital anomalies [11]. In addition, the history of occupational exposure of fathers of children with cancer with EMFs greater than 2 mG during the pre-conceptual period was not associated with a significant increase in cancer in their children [40]. But the risk of ALL cancer in children who lived near high-voltage power lines at a distance of fewer than 600 m or for more than 4 years during prenatal and postnatal periods increased by 3.65 times, which was the most important risk factor among the 11 risk factors of this disease [30,76]. Furthermore, regarding the role of maternal exposure to RF-EMFs on childhood neurodevelopment, it has been shown that speech problems were significantly higher in children whose mothers used more cordless telephones before and during pregnancy, as well as those who lived near high-voltage power lines before and during pregnancy [38]. The weaknesses of two recent studies were that the exact extent of exposure to EMFs and even the living distance of mothers during pregnancy and postpartum with the electric flux of EMFs of high-voltage power lines were not determined [38]. Furthermore, the assessment of the mother’s exposure was frequently done through a retrospective questionnaire, which is more likely to recall bias [40].

The results of the present meta-analysis showed that children whose parents were exposed to various types of EMFs (ionized and non-ionized) were 14 times more likely to develop cancers (such as rhabdomyosarcoma, ALL, brain tumors, and neuroectoderm). ALL is the most common blood malignancy in childhood and accounts for a quarter of all childhood cancers [77]. The results of a study showed that the residence period of more than 4 years near high-voltage power lines (producing ELF-EMFs) before and after birth is an important risk factor for ALL in childhood [30]. Other studies have also shown that occupational exposure of mothers to ELF-EMFs is associated with an increased risk of leukemia [78] and brain tumors [79]. In addition, chronic exposure children under 15 years of age with RFR From radio transmitters 31 AM amplitude modulation with a power of 20 kW or more, although not associated with an increase in brain cancer and infantile, lymphocytic leukemia in adolescents increases significantly and the risk ratio increases with distance from the radio transmitter source less. It is connected from 2 km [39]. The strength of this population-based study was that exposure to RFR using an RFR exposure prediction program from AM transmitter transmitters based on the flat-earth attenuation model, which was verified by real measurement.

Due to the importance of genome cell vulnerability and the possibility of sperm genetic alteration against EMFs, the results of a study showed that fathers’ occupational exposure to ELF-EMFs greater than 0.2 μT (2 mG) [40] in the preconception period was not associated with an increase in cancers in their offspring. More than 0.4 μT (4 mG) is associated with an increased incidence of neuroblastoma. However, in this study, information about parents’ exposure to ELF-EMFs was very limited [42]. Possible mechanisms include changes in the mechanical function of cellular proteins, ion channels, membrane receptors, and enzymes, which vary in frequency depending on the cell type and duration of exposure [80].

Fetal exposure to X-rays during perinatal is one of the few environmental risk factors for childhood cancer. The results of some studies have shown that mothers’ exposure to X-rays throughout pregnancy (pelvimetry), repeated ultrasounds, and parental exposure to preconception, as well as postpartum exposure, are not associated with enhancement of risk of ALL and brain tumors. However, the same studies have shown that mothers’ exposure to X-rays during pregnancy particularly increases the chances of developing ALL pre-B cell forms and primitive neuroectodermal brain tumors (PNET). The origin of PNET is from undifferentiated neurons [56]. It is supposed that exposure of stem cells to X-rays during their differentiation leads to genome instability, cumulative DNA damage, histone methylation, and DNA changes that can lead to malignancy [81]. Considering that the “vulnerability window” of the cell is mainly during the first trimester. It is important to mention that in these studies, most of the X-ray examinations of mothers were performed in the first trimester. The results of another study revealed that performing any examination (abdominal and non-abdominal) with X-rays during the whole pregnancy, especially the first trimester, increases the risk of embryonal RMS fetal rhabdomyosarcoma [41]. It seems that the risk of rhabdomyosarcoma associated with X-rays during pregnancy is somewhat higher than that reported for leukemia and other solid tumors.

### 4.1 Strong and limitations

This study was the first report about the effect of EMF on fetal and childhood abnormalities, including comprehensive search, using complex statistical method to pool data and evaluation of the source of heterogeneity were the current study’s strengths. Similar to other studies, our research had the following limitations: (1) Studies, in addition to examining various tools, examined the extent of different exposures to multiple outcomes. Although we wanted to examine the effect of different levels of exposure, it was not possible to extract such data from the articles. (2) We had a tendency to estimate the pooled SMD in different geographical regions or regional-specific estimation based on available methods [82]. Due to the insufficient number of studies in this field, this estimation will not be robust.

## 5 Conclusion

Studies are associated with mothers’ exposure to EMFs during pregnancy and non-ionizing radiation (RF and ELF-EMFs) with fetal complications such as significant enhancement of oxidant factors, decrease of antioxidant factors, and increase in DNA damage parameters, as well as changes in expression proteins in cord blood genes. On the other hand, close maternal exposure in prenatal and postnatal (residence or occupational exposure) with EMFs of high voltages power lines more than 1 mG or 50 Hz with congenital anomalies (CNS defect, spina bifida) and fetal developmental disorders (such as reduced embryonic bud length) and neurodevelopmental disorders in childhood (e.g., speech problems in children) are associated. Moreover, parents’ exposure to ionizing and non-ionizing radiation (X-ray, ELF-EMF, and RF examinations) before and after birth is also associated with enhancement of the risk of cancers (such as rhabdomyosarcoma, ALL, brain tumors, and neuroectoderm) in childhood and adolescence. However, due to the limitations of studies, such as inaccurate measurement of exposure to ELF-EMF (e.g., interviews based on participants’ reminders) or inaccurate measure of the actual rate of exposure to EMF or case–control model of most studies, the effects of EMF on fetal and childhood abnormalities should be interpreted with caution. Considering the widespread exposure to non-ionizing radiation, a little enhancement of exposure to EMF could lead to unacceptable health consequences for future generations. Although the number of epidemiological studies examining the undesirable effects of EMF exposure in humans is limited, the findings of this study should prompt further research on this significant environmental danger for pregnant women.

## Abbreviations

ALL: Acute lymphocytic leukemia
AM: Amplitude modulation
ELF-EMF: Extremely low-frequency-electromagnetic fields
EMFs: Electromagnetic fields
GHz: Giga Hertz
HVACPL: High-voltage alternating current power transmission lines
Hz: Hertz
mG: mill gauss
MHz: Mega Hertz
MRI: Magnetic resonance imaging
RF: Radio frequency
RFR: Radio frequency radiation
TV: Television

## Appendix

**Box A1:** detail of developed search strategy based on Medline data base

((((((embryonic disorder[MeSH Terms]) OR (embryonic disorder[Title/Abstract])) OR ((fetal disorder[Title/Abstract]) OR (fetal disorder[MeSH Terms]))) OR ((childhood abnormality[MeSH Terms]) OR (childhood abnormality[Title/Abstract]))) OR ((childhood disorder[Title/Abstract]) OR (childhood disorder[MeSH Terms]))) AND (((((Childhood[MeSH Terms]) OR (Childhood[Title/Abstract])) OR ((Fetal[Title/Abstract]) OR (Fetal[MeSH Terms]))) OR ((Fetus[MeSH Terms]) OR (Fetus[Title/Abstract]))) OR ((embryonic[Title/Abstract]) OR (embryonic[MeSH Terms])))) AND ((((((Cell Phone[Title/Abstract]) OR (Cell Phone[MeSH Terms])) OR ((Electromagnetic Fields[MeSH Terms]) OR (Electromagnetic Fields[Title/Abstract]))) OR ((Electromagnetic Radiation[Title/Abstract]) OR (Electromagnetic Radiation[MeSH Terms]))) OR ((Electromagnetic Wave[MeSH Terms]) OR (Electromagnetic Wave[Title/Abstract]))) OR ((Electromagnetic Energy[Title/Abstract]) OR (Electromagnetic Energy[MeSH Terms])))

**Box A2:** detail of developed search strategy based on Scopus data base

(TITLE-ABS (fetal AND disorder) OR TITLE-ABS (fetus AND disorder) OR TITLE-ABS (fetus AND abnormality) OR TITLE-ABS (fetus AND abnormality) OR TITLE-ABS (embryonic AND disorder) OR TITLE-ABS (embryonic AND abnormality) OR TITLE-ABS (childhood AND abnormality) OR TITLE-ABS (childhood AND disorder) OR TITLE-ABS (fetal AND disease) OR TITLE-ABS (embryopathies) OR TITLE-ABS (embryopathy)) AND (TITLE-ABS (electromagnetic AND field) OR TITLE-ABS (electromagnetic AND fields) OR TITLE-ABS (electromagnetic AND wave) OR TITLE-ABS (electromagnetic AND waves) OR TITLE-ABS (cellphone) OR TITLE-ABS (x-ray) OR TITLE-ABS (video AND display AND terminals) OR TITLE-ABS (microwave) OR TITLE-ABS (microwaves) OR TITLE-ABS (electromagnetic AND energy) OR TITLE-ABS (cellular AND phone) OR TITLE-ABS (cellphone) OR TITLE-ABS (mobile AND phone))
